# The sex difference in self-rated health among older Turkish and Moroccan migrants in the Netherlands: an exploratory study of contributing determinants

**DOI:** 10.1186/s12889-023-17479-6

**Published:** 2024-01-22

**Authors:** Lena D. Sialino, H. A.H. Wijnhoven, S. H. van Oostrom, H. S.J. Picavet, M. Visser, L. A. Schaap

**Affiliations:** 1 Department of Health Sciences, Faculty of Science, Amsterdam Public Health research institute, Vrije Universiteit Amsterdam, Amsterdam, the Netherlands; 2 Centre for Nutrition, Prevention and Health Services, National Institute for Public Health and the Environment, Bilthoven, the Netherlands

**Keywords:** Gender differences, Dutch migrants, Healthy ageing, Lifestyle factors, Health factors, Socio-demographic factors, Social factors, Intersectionality

## Abstract

**Background:**

Although being a woman and having a migration background are strong predictors of poor self-rated health among (older) adults, research on the sex difference in self-rated health among (older) migrants remains limited. This study therefore aims to investigate this topic and explore the contributing role of determinants of self-rated health.

**Methods:**

Cross-sectional data from 360 Turkish-Dutch and Moroccan-Dutch adults aged 55–65 as part of the Longitudinal Aging Study Amsterdam (LASA) were used. Self-rated health (good versus poor) was measured by a single item question. Univariate age-adjusted logistic regression analysis was used to investigate the sex difference in self-rated health and the contribution of sex differences in sensitivity (strength of the association) and/or exposure (prevalence) to socio-demographic, social, lifestyle or health-related determinants of self-rated health.

**Results:**

Women had a 0.53 times lower odds (95%CI:0.40–0.82, p = 0.004) on good self-rated health compared to men. Women more often having a lower education level, living alone and having a higher prevalence of depressive symptoms, chronic diseases and especially functional limitations contributed to the lower self-rated health among women. In contrast, men were more sensitive to the impact of memory complaints, depressive symptoms, visual difficulties and functional limitations.

**Conclusions:**

Older Turkish-Dutch and Moroccan-Dutch women have a significant lower self-rated health compared to men. Women having a higher exposure to both socio-demographic and health-related determinants of self-rated health, which contributed to the sex difference. Future research should take these differences in self-rated health and determinants between women and men into account when investigating health among older migrants.

**Supplementary Information:**

The online version contains supplementary material available at 10.1186/s12889-023-17479-6.

## Background

Due to an increase in life expectancy in the past century, ageing has become a key demographic trend of developed country populations. Migration and feminization (i.e. larger proportion of women among older adults) also shape the population, demonstrating the need for health research focusing on older women and migrants. In general, studies demonstrated that older women and older adults with a migration background experience poorer health, including self-rated health, compared to respectively men and the indigenous population [1–5].

Self-rated health forms an important and frequently studied aspect of health, since it encompasses one’s individual assessment of general health. It is shown to be a strong predictor of mortality, morbidity and disability [6–8], and can sometimes predict mortality better than objective measures of health as it also captures other individual aspects such as mental, social and psychological health [7, 9, 10]. As Mladovsky et al. (2009) stated: “Self-rated health is not only a function of actual health status, but also of an individual’s perception of health.” [11].

Being a woman and having a migration background are both known predictors of poor self-rated health among adults worldwide [2, 4, 5]. Additionally, some studies suggest that sex difference in self-rated health (to the disadvantage of women) are larger among the migrant populations compared to the indigenous population [12–16]. For example, in the Netherlands, women aged 18 years and older with a Turkish or Moroccan migration background had a respectively 2.72 (95%CI:1.76–4.18) and 1.95 (95%CI:1.27–3.01) times higher odds on poor self-rated health compared to men, while this was only 1.14 times higher odds (95%CI:1.01–1.29) among indigenous Dutch adults [14, 15]. There is, however, limited research on older (migrant) adults specifically. Among older indigenous populations, the sex difference in self-rated health seems to decrease or even diminish at older ages (55 years and older) [17, 18]. Whether this is also the case among older migrants remains unstudied, except for one recent study among Turkish migrants in Germany that showed that women have a lower self-rated health compared to men [16]. Previously, studies focusing on migrant differences in self-rated health have adjusted for sex [3, 19, 20] and studies focusing on sex differences in self-rated health are limited to the (older) adult indigenous population [18, 21, 22]. Therefore, there is a clear need for research combining the focus on sex difference among migrant (older) adults. In the Netherlands, Turkish and Moroccan migrants form the two largest non-western first-generation migration groups and are therefore an important group currently ageing in the Netherlands [23].

It has been demonstrated that social, lifestyle, health-related and especially socio-economic determinants contribute to the self-rated health of migrant groups worldwide [11, 24, 25]. Although it is also known that these determinants can differ between migrant men and women [16, 25], their contribution to the observed sex difference in self-rated health remains unstudied. The identification of such contributing determinants will provide a better understanding of the sex difference in self-rated health among older migrants and may provide the first insights for developing sex-specific prevention strategies to enhance (self-rated) health among older ethnic minorities.

This study aims to investigate the sex difference in self-rated health among a sample of Turkish-Dutch and Moroccan-Dutch older adults aged 55–65 years in the Netherlands and explores the contributing role of socio-demographic, social, lifestyle and health-related determinants of self-rated health in this difference in self-rated health.

## Methods

### Study population

Cross-sectional cohort data from the Longitudinal Aging Study Amsterdam (LASA) were used [26]. LASA is a long-term ongoing cohort study initiated to determine predictors and consequences of ageing. In 2013–2014 a cross-sectional migration cohort was included of Turkish and Moroccan first-generation migrants aged 55–65 years and living in the Netherlands (n = 478). This cross-sectional migration cohort was the study population of the current study. Data collection of this migration cohort took place in 15 Dutch cities with population sizes between 85,000 and 805,000 inhabitants (Amsterdam, Zwolle, Oss, Alkmaar, Almere, Amersfoort, Breda, Eindhoven, Enschede, Haarlem, Helmond, Hilversum, Nijmegen, Tilburg and Zaanstad), because Turkish and Moroccan migrants predominantly live in urban areas. Trained interviewers of the same ethnic background conducted face-to-face interviews in Turkish, Moroccan Arabic (Darija) or Berber language (Tarifit). If questionnaires were not available in Moroccan Arabic, Berber or Turkish, questions were translated by two professional translators according to the back-and-forth method. All questionnaires were evaluated and tested in pilot-interviews. To be able to compare univariate regression analysis across different determinants (see statistical analysis paragraph) only participants with data on all variables were included, leading to an analytic sample of n = 360 (out of the n = 478 study population). For a more detailed description of LASA and the (translated) questionnaires, see Hoogendijk et al. (2019) [26]. Ethical approval for the LASA study was given by the Medical Ethics Committee of the VU University Medical Centre Amsterdam, and all participants provided written informed consent.

### Self-rated health

In this study we employ the widely used self-rated health measurement of the general health question of the Short Form-36 (SF-36), validated among older adults and ethnic groups [27]. Respondents were directly asked to rate their health with a single question; “How would you rate your current health?”. The responses originally given on a five-point Likert scale were dichotomized into good (1; excellent, good and ok) and poor (0; sometimes good and sometimes poor, poor) self-rated health [11, 14, 28].

### Socio-demographic determinants

Age was based on the time between the date of the interview and the birth date, expressed in years. Sex was defined as man or woman, as determined at birth. Education was categorized into low (elementary education or less), middle (lower vocational education and general intermediate education) and high educational level (intermediate vocational education, general secondary education, higher vocational education, college education and university). Living situation was assessed by asking the question “Next to yourself, how many other persons are part of your household?” and dichotomized as “living alone” or “living with one or more other persons”.

### Social determinants

Frequency of mosque attendance was assessed with the question: “Do you attend mosque services or meetings of your religious group, and if so, how often?”. The responses originally given following a 6-point scale were dichotomized into “often” (once a week or more) or “never to sometimes” (never to a maximum of three times per month). To assess the degree of participation in societal activities, the respondent was shown a list of organizations or associations such as a political organization, association for older adults or religious society, and asked to indicate of which (s)he was a member, and whether (s)he visits meetings or activities of the organization or association, dichotomized into “no” (inactive) and “yes” (active). Religious coping was assessed by several abbreviated versions of the Religious Coping Scale and consisted of three items: two positive items (I turn to God for strength, support and guidance in crises situations, and, I confess my sins and ask for God’s forgiveness) and one negative item (I wonder if God has abandoned me). The answer categories ranged from “never” (0) to “very often” (3) and were dichotomized into “often to very often” (6–9) and “never to sometimes” (0–5). Loneliness was assessed using the translated 11-item De Jong Gierveld scale ranging from 0 (no loneliness) to 11 (severe loneliness). Social contact frequency was assessed using the following phrase: “How often do you have contact with children, grandchildren, children-in-law, uncles/aunts/siblings/in-laws, Moroccan or Turkish friends/acquaintances, Dutch friends/acquaintances, Moroccan or Turkish neighbors and Dutch neighbors. Contact means that you visit them or they visit you, or have contact by phone, writing, or email.” The five answer categories ranged from “daily” to “never or less than once a year”. The total score ranged from zero to thirty-two.

### Lifestyle determinants

Physical activity was assessed by asking how often and for how long participants performed each of the following activities in the past two weeks: walking outdoors, biking, heavy household activities and the most intensive sport activity they performed. It was defined as total MET hours/week of physical activity.

### Health determinants

Memory complaints were assessed by the question “Do you have complaints about your memory?” with the answering options “yes” and “no”. Depressive symptoms during the past week were assessed using the translated 20-item Centre for Epidemiologic Studies Depression Scale (CES-D), ranging from 0 (rarely or none of the time; less than one day per week) to 3 (most or almost all the time; five–seven days per week), transformed into a score ranging from 0 to 60. Visual and hearing difficulties were assessed by the question “Can you see/hear well enough” with four answer categories: “yes, without difficulties”, “yes, but with some difficulty”, “yes, with major difficulty” and “no, I cannot”. Both were dichotomized into “having difficulties” and “not having difficulties”. Self-reported chronic diseases were defined as the number of chronic diseases of the most frequently occurring somatic chronic diseases in the Netherlands: chronic non-specific lung disease, cardiac disease, peripheral artery diseases, diabetes mellitus, cerebrovascular accident or stroke, osteoarthritis, rheumatoid arthritis and/or cancer and a maximum of two other chronic diseases which symptoms lasted for at least three months. The total score was categorized into four groups ranging from “no chronic disease” to “three of more chronic diseases”. Functional limitations were assessed by using a validated questionnaire concerning the degree of difficulty with performing the following six activities of daily living: climbing stairs, walking 5 min outdoors without resting, getting up and sitting down in a chair, dressing and undressing oneself, using own or public transportation, and cutting one’s own toenails. The five response categories ranged from “no, I cannot” to “yes, without difficulty” where the number of items were counted and categorized into “no difficulties”, “some difficulties” and “a lot of difficulties”.

### Statistical analyses

Logistic regression analysis with good self-rated health as dependent variable was used to investigate the sex difference in self-rated health and sex differences in sensitivity and exposure to determinants of self-rated health. Variables with more than 30% missings were excluded (alcohol consumption, smoking, BMI, anxiety and pain). Each univariable association between the determinant and self-rated health was investigated and reported for men and women separately. To increase our power in these univariate analysis, Turkish-Dutch and Moroccan-Dutch older adults were analyzed together. In order to test the sex differences in sensitivity, we examined the interaction term determinant*sex separately for each determinant in an univariate age-adjusted model. A p < 0.10 belonging to this interaction regression coefficient was considered as a statistically significant difference in sensitivity, i.e. the strength of the association between the determinant and self-rated health. In order to evaluate the sex difference in exposure (i.e. prevalence) we determined the percentage change of the regression coefficient of the variable sex in the model with self-rated health after adjustment for the determinant of interest [29]. A relevant percentage change was set at > 10% to detect a change in this relatively small study sample.

## Results

### Study population

The study population (n = 360) consisted of 220 men (61%) and 140 women (39%) (Table 1). Men and women were comparable in mean age (61 years, SD approximately 3.0) and migration background (approximately 60% Turkish and 40% Moroccan). Compared to men, women were lower educated, more often lived alone, less often visited a mosque, were less often active member of an organization, had a lower prevalence of good self-rated health and higher prevalence of depressive symptoms, visual difficulties, chronic diseases and functional limitations. When compared to the total study population of the migration cohort from LASA (n = 478), the analytical sample showed similar baseline characteristics (supplementary Table 1).

**Table 1:** Characteristics for men and women of the study population

Characteristics	Study population (n = 360)		
	Women (n = 140, 39%)	Men (n = 220, 61%)	
*Sociodemographic*			
Age in years (range 55–65 years)	61 (2.9)	61 (3.0)	
Migration background			
Turkish	85 (61%)	127 (58%)	
Moroccan	55 (39%)	93 (42%)	
Education level			
Low	114 (81%)	141 (66%)	
Middle	15 (11%)	37 (17%)	
High	11 (8.0%)	42 (19%)	
Living situation (alone)	44 (31%)	28 (13%)	
*Social*			
Mosque visit			
Never to max 3 times a month	84 (60%)	50 (23%)	
At least once a week	56 (40%)	170 (77%)	
Societal activities participation			
Active in at least one	56 (40%)	171 (77%)	
Not active	84 (60%)	49 (23%)	
Religious coping			
Never to sometimes	60 (43%)	122 (55%)	
Often to very often	80 (57%)	98 (45%)	
Loneliness (0–11)	5.1 (2.9)	5.1 (3.3)	
Social contact frequency (0–32)	20 (5.2)	20 (5.9)	
*Lifestyle*			
Physical activity (Meth/w)	25 (35)	26 (38)	
*Health-related*			
Self-rated health (good)	49 (35%)	111 (51%)	
Memory complaints (yes)	66 (47%)	99 (45%)	
Depression symptoms (0–60)	19 (12)	16 (10)	
Visual difficulties (yes)	67 (48%)	81 (37%)	
Hearing difficulties (yes)	38 (27%)	61 (27%)	
Chronic diseases (n)			
None	20 (15%)	61 (28%)	
1	35 (25%)	63 (29%)	
2	43 (31%)	45 (21%)	
3 or more	42 (30%)	51 (23%)	
Functional limitations			
None	25 (18%)	87 (40%)	
Some	37 (26%)	68 (31%)	
A lot	78 (56%)	65 (30%)	

### Sex difference in self-rated health

There was a significant sex difference in self-rated health among adults aged 55–65 years with a Turkish or Moroccan migration background. In total 35% (n = 49) of the women rated their own health as good versus 51% (n = 111) of the men. Logistic age-adjusted regression analyses demonstrated that women had a 0.53 times lower odds (95%CI: 0.40–0.82, p = 0.004) on good self-rated health compared to men. A similar sex difference was found when using data of self-rated health of the whole study population of the migration cohort (0.53 times lower odds, 95%CI:0.36–0.76, p < 0.001, n = 472).

### Sex difference in sensitivity to determinants of self-rated health

In general, men and women had similar determinants of self-rated health: low educational level, loneliness, depressive symptoms, chronic diseases and functional limitations were associated with a lower odds of having good SRH (p < 0.05) (Table 2). Only for several health-related determinants the strength of the association with self-rated health (i.e. sensitivity) differed significantly between women and men (interaction term p < 0.10): Memory complaints, depressive symptoms, visual difficulties, one versus no chronic disease and functional limitations. The association between having one versus none chronic disease with self-rated health was stronger among women compared to men (p = 0.070) (Table 2), but no trend was observed for more than one chronic disease. The association between having memory complaints (p = 0.027), having more depressive symptoms (p = 0.001), having visual (p = 0.077) and functional limitations (p = 0.032) with self-rated health was stronger among men compared to women, meaning men were more sensitive for these determinants (Table 2). To illustrate: men with a lot of functional limitations had a 0.041 times lower odds (95%CI: 0.02–0.10, p < 0.05) on good self-rated health compared to men without functional limitations, while for women this was a 0.167 times lower odds (95%CI: 0.06–0.45, p < 0.05).

**Table 2:** Determinants of self-rated health by sex and sex difference in the sensitivity to determinants of self-rated health

Variable	Univariate logistic regression model				
	**Women (n = 140)**		**Men (n = 220)**		***P-value*** interaction variable*sex
	OR *	95% CI	OR *	95% CI	
Raw model (Age)	0.963	0.85 ─ 1.09	0.977	0.90 ─ 1.07	0.843
*Sociodemographic*					
Education level					
Middle vs. low	1.041	0.33 ─ 3.27	**2.214**	1.03 ─ 4.77	0.292
High vs. low	**3.573**	0.98 ─ 13.0	1.454	0.72 ─ 2.92	0.218
Living situation (alone)	**0.431**	0.19 ─ 0.97	0.596	0.27 ─ 1.34	0.579
*Social*					
Mosque visit (regularly)	0.798	0.39 ─ 1.63	**0.493**	0.27 ─ 0.95	0.329
Societal participation (inactive)	0.740	0.36 ─ 1.50	0.802	0.42 ─ 1.53	0.853
Religious coping (often)	1.323	0.65 ─ 2.70	0.726	0.43 ─ 1.24	0.193
Loneliness (0–11)	**0.880**	0.78 ─ 0.99	**0.845**	0.78 ─ 0.92	0.596
Social contact frequency (0–32)	0.981	0.92 ─ 1.05	1.03	0.98 ─ 1.08	0.231
*Lifestyle*					
Physical activity (Meth/w)	1.010	0.99 ─ 1.02	1.01	1.00 ─ 1.02	0.780
*Health-related*					
Memory complaints (yes)	0.502	0.24 ─ 1.03	**0.179**	0.10 ─ 0.32	**0.027**
Depressive symptoms (0–60)	**0.955**	0.92 ─ 0.98	**0.870**	0.83 ─ 0.91	**0.001**
Visual difficulties (yes)	0.756	0.37 ─ 1.53	**0.329**	0.19 ─ 0.58	**0.077**
Hearing difficulties (yes)	0.482	0.21 ─ 1.13	**0.240**	0.13 ─ 0.46	0.203
Chronic diseases (n)					
1 vs. none	**0.223**	0.07 ─ 0.73	0.775	0.37 ─ 1.64	**0.070**
2 vs. none	**0.230**	0.07 ─ 0.72	**0.222**	0.10 ─ 0.51	0.979
3 or more vs. none	**0.101**	0.03 ─ 0.35	**0.165**	0.07 ─ 0.38	0.484
Functional limitations					
Some vs. none	0.382	0.13 ─ 1.09	**0.178**	0.09 ─ 0.37	0.235
A lot vs. none	**0.167**	0.06 ─ 0.45	**0.041**	0.02 ─ 0.10	**0.032**

* OR on good self-rated health. OR < 1 is lower odds on good self-rated health and OR > 1 is higher odds on good self-rated health

*Note*: bold indicates significant (variable *p* < 0.05, interaction term *p* < 0.10)

### Sex difference in exposure to determinants of self-rated health

For most determinants of self-rated health men and women differed in exposure. Women were more exposed to low educational level, living alone, depressive symptoms, visual difficulties, chronic diseases and functional limitations (Table 1). When adjusting for these determinants in a univariate model, the sex difference in self-rated health significantly decreased (> 10%) for: educational level (-14%), living situation (-18%), depressive symptoms (-25%), chronic disease (-31%) and functional limitations (-84%) (Table 3). So, women more often being exposed to these determinants of self-rated health significantly contributed to the sex difference in self-rated health. To illustrate: when adjusting for educational level, the sex difference in self-rated health decreased with 14% (from − 0.641 to -0.550). In contrast, men were more exposed to regular mosque visit (Table 1), a significant determinant of poor self-rated health among men (Table 2). When adjusted for mosque visit, the sex difference in self-rated health significantly increased (+ 20%) (Table 3).

**Table 3:** Sex difference in exposure to determinants of self-rated health

Variable	Beta sex *	95% CI	% Change Beta sex **
Raw model (Age)	**−0.641**	−1.08 ─ −0.21	*Reference*
*Sociodemographic*			
Education level (high/middle vs. low)	**−0.550**	−1.00 ─ −0.10	**−14%**
*Social*			
Living situation (together vs. alone)	**−0.528**	−0.98 ─ −0.08	**−18%**
Mosque visit (regular vs. some to never)	**−0.832**	−1.31 ─ −0.35	**19%**
Societal participation (inactive versus active)	**−0.688**	−1.14 ─ −0.24	−7.3%
Loneliness (0–21)	**−0.684**	−1.13 ─ −0.23	6.6%
Religious coping (often vs. some to never)	**−0.629**	−1.07 ─ −0.19	−2.0%
Social contact frequency (0–32)	**−0.642**	−1.08 ─ −0.20	0.2%
*Lifestyle*			
Physical activity (Meth/w)	**−0.636**	−1.08 ─ −0.19	−0.8%
*Health-related*			
Memory complaints (yes vs. no)	**−0.679**	−1.14 ─ −0.22	5.9%
Depression symptoms (0–60)	**−0.483**	−0.96 ─ −0.00	**−25%**
Visual difficulties (yes vs. no)	**−0.585**	−1.02 ─ −0.13	−0.9%
Hearing difficulties (yes vs. no)	**−0.686**	−1.14 ─ −0.24	7.0%
Chronic diseases (vs. none)	−0.443	−0.91 ─ 0.02	**−31%**
Functional limitations (vs. none)	−0.105	−0.61 ─ 0.40	**−84%**

* Natural exponent of the sex difference (women versus men) in odds on good self-rated health

** Percentage change = 1 – (Beta sex univariate model including the variable / Beta sex raw model)

*Note*: Bold is significant; *p*-value Beta sex < 0.05

*Note*: Univariate analysis; each row represents the raw model including the corresponding variable

*Note*: A negative percentage change means this variable causes a decrease of the sex difference when adjusted for, so partly explains it

*Note*: A positive percentage change means this variable causes an increase of the sex difference when adjusted for, so partly suppresses it

## Discussion

The findings of this study demonstrate a significant sex difference in self-rated health among a sample of older Turkish-Dutch and Moroccan-Dutch adults (aged 55–65 years), to the disadvantage of women. In general, men were more sensitive for health-related determinants of self-rated health and women were more exposed to socio-demographic and health-related determinants of self-rated health. The latter contributed significantly to the observed lower odds on good self-rated health among older migrant women.

The observed lower self-rated health among Dutch older migrant women in this study adds to current research on sex differences in the health of migrants—which largely focuses on younger population groups—with evidence showing that women in older migrant populations are at a health disadvantage [4, 12–16, 24, 28, 30, 31]. The sex difference of 0.53 times lower odds on good self-rated health among older migrant women in this study is comparable to the sex difference found in another Dutch study among younger migrant adults, ranging from 0.37 to 0.52 [14]. The sex difference among migrants therefore does not seem to decrease from the age of 55 years and older, which has been suggested for the indigenous Dutch population [17, 18]. A study among the indigenous Turkish population showed that the sex difference in self-rated health increases with age (when comparing 35–44 years with 45–54 and 55–64 years) [32] and was absent among the indigenous Moroccan population (aged 60 years and older) [33]. Future research including a larger age range (65 years and older), preferably using longitudinal data, could determine how the sex difference develops at older ages among migrant populations.

The results of this study confirmed several socio-demographic (educational level and living situation) and health-related determinants (loneliness, depressive symptoms, chronic diseases and functional limitations) of self-rated health among older migrants, in line with previous literature [11, 25]. For example, a recent Italian study showed that determinants related to economic position were positively associated with self-rated health among older migrant adults [11], which relates to the reported positive association with educational level in our study. Although migrants are known to be at risk for low physical activity [34] and it has been shown to effect mental and physical health [35], it was not associated with self-rated health in our study.

This study is the first to investigate differences in sensitivity to determinants of self-rated health among older migrant adults. Migrant older men were found to be more sensitive to several health-related determinants: memory complaints, depressive symptoms and visual and functional limitations. Although depressive symptoms and functional limitations are more common among older migrant women, if men are exposed to these, it seems that the negative impact on self-rated health is larger. This might reflect differences in health care utilization, where migrant men seek less help for mental and physical health problems compared to women [14, 36]. However, it might also reflect gender differences in the perceived burden of health-related determinants or in the report of self-rated health. Indeed, an older study has demonstrated that older women are less likely to assess their health as being poor than men of the same age for the same level of functional disability [37].

The results of this study confirm a higher exposure to several socio-demographic (educational level and living alone) and health-related determinants (depression symptoms, chronic diseases and functional limitations) among women compared to men. Furthermore, it demonstrates that this contributes significantly to the observed lower odds on good self-rated health among older migrant women. This is in line with a recent study demonstrating that older women with a Turkish migration background in Germany form a high risk group for poor self-rated health, which was mediated by emotional loneliness and especially physical limitations [16]. Although loneliness on the Gierveld scale did not significantly contribute in our study, living alone and depressive symptoms did, which may represent an indication of emotional loneliness. To the best of our knowledge, similar studies among moroccan older migrants have not been conducted. However, another recent study among migrant older adults in China also demonstrated the mediating effect of living alone on the lower self-rated health among women compared to men [38]. The higher prevalence of chronic diseases and lower educational level among migrant older women has been often demonstrated in literature [14, 39, 40], but its contributing effect on the sex difference in health was demonstrated in this study for the first time. Very frequent visiting of the mosque was associated with a lower odds on good self-rated health among men. This could be related to the reason they are frequent visitors, which might be because of mental of health problems or a small indigenous social network, both associated with a lower self-rated health [11]. However, additional research in multivariable models is needed to better understand this observation.

The current study has several strengths. First, the measurement of self-rated health is a widely used indicator of self-assessed health and validated among middle-aged and ethnic groups [11, 27]. Secondly, this study is, to the best of our knowledge, the first to systematically investigate the contributing role of a wide range of determinants of self-rated health in the female disadvantage in self-rated health among older migrants. A limitation of this study is the low population sample size, which limited our analyses to univariate models. Furthermore, data on some known determinants of self-rated health were lacking due to missing values or exclusion from data collection [28], such as lifestyle factors and perceptions on ageing [41, 42]. Also, due to statistical power limitations, the Turkish-Dutch and Moroccan-Dutch migrants were pooled. However, it provides some essential new insights in the contributing roles of determinants among this difficult to reach and understudied population using two different approaches (sensitivity and exposure). Future research should take a more extensive range of lifestyle-related determinants into account using a multivariable model and investigate possible differences between older Turkish-Dutch and Moroccan-Dutch older migrants.

Our study results suggest that policies aiming to improve self-rated health among Turkish-Dutch and Moroccan-Dutch older women requires more focus on socio-demographic and health-related determinants, compared to men, especially physical limitations. It also suggests that (although a small group compared to women) older Turkish-Dutch and Moroccan-Dutch migrant men with health problems might be an important target group for improving self-rated health due to their higher sensitivity. Overall our study shows that future research investigating health among older migrants should take differences in sensitivity and exposure between women and men into account.

## Conclusions

Turkish-Dutch and Moroccan-Dutch women aged 55–65 years have a significant lower self-rated health compared to men; a 0.53 times lower odds on good self-rated health compared to men. Women having a higher exposure to both socio-demographic and health-related determinants of self-rated health contributed to this sex difference in self-rated health. In particular, the higher exposure to physical limitations among older migrant women compared to men. Future research and policy and practice should take these differences in self-rated health and determinants between women and men into account when respectively investigating or aiming to improve health among older migrants.

### Electronic supplementary material

Below is the link to the electronic supplementary material.

Supplementary Material 1

## Data Availability

Data cannot be shared publicly because of confidentiality. Data are available from the LASA Institutional Data Access / Ethics Committee (contact via https://www.lasa-vu.nl/index.htm) for researchers who meet the criteria for access to confidential data. The data underlying the results presented in the study are available from the Longitudinal Aging Study Amsterdam (https://www.lasa-vu.nl/index.htm). The LASA Steering Group will review all requests for data to ensure that proposals for the use of LASA data do not violate privacy regulations and are in keeping with informed consent that is provided by all LASA participants. The authors of this study do not have any special access privileges to the data underlying this study that other researchers would not have.
