# Supplementary material for: The sex difference in self-rated health among older Turkish and Moroccan migrants in the Netherlands: an exploratory study of contributing determinants
Source: BMC Public Health. 2024 Jan 22;24:248. doi: 10.1186/s12889-023-17479-6 (PMC10801924; doi:10.1186/s12889-023-17479-6)
Supplement: Supplementary file 1 — Supplementary Material 1 [file 12889_2023_17479_MOESM1_ESM.docx]

**Supplementary Table 1 Characteristics for men and women of the study population and total LASA migration cohort.**

| Characteristics | Study population  (n = 478) | | Analytical sample  (n = 360) | |
| --- | --- | --- | --- | --- |
|  | Women  (n=204, 43%) | Men  (n=268, 57%) | Women  (n=140, 39%) | Men  (n=220, 61%) |
| *Socio-demographic* |  |  |  |  |
| Age in years (range 55-65) | 61 (2.9) | 61 (3.0) | 61 (2.9) | 61 (3.0) |
| Migration background |  |  |  |  |
| Turkish | 121 (60%) | 148 (54%) | 85 (61%) | 127 (58%) |
| Moroccan | 82 (40%) | 127 (46%) | 55 (39%) | 93 (42%) |
| Education level |  |  |  |  |
| Low | 164 (83%) | 179 (65%) | 114 (81%) | 141 (66%) |
| Middle | 19 (9.5%) | 45 (17%) | 15 (11%) | 37 (17%) |
| High | 15 (7.5%) | 50 (18%) | 11 (8.0%) | 42 (19%) |
| Living situation (alone) | 66 (33%) | 40 (15%) | 44 (31%) | 28 (13%) |
| *Social* |  |  |  |  |
| Mosque visit |  |  |  |  |
| Never to max 3 times a month | 110 (58%) | 52 (20%) | 84 (60%) | 50 (23%) |
| At least once a week | 80 (42%) | 212 (80%) | 56 (40%) | 170 (77%) |
| Societal participation (not active) | 99 (55%) | 63 (20%) | 84 (60%) | 49 (23%) |
| Religious coping |  |  |  |  |
| Never to sometimes | 88 (44%) | 126 (50%) | 60 (43%) | 122 (55%) |
| Often to very often | 113 (56%) | 124 (50%) | 80 (57%) | 98 (45%) |
| Loneliness (0-11) | 5.2 (3.2) | 5.1 (3.3) | 5.1 (2.9) | 5.1 (3.3) |
| Social contact frequency (0-32) | 19 (6.0) | 19 (5.3) | 20 (5.2) | 20 (5.9) |
| *Lifestyle* |  |  |  |  |
| Physical activity (Meth/w) | 25 (36) | 27 (38) | 25 (35) | 26 (38) |
| *Health-related* |  |  |  |  |
| Self-rated health (good) | 73 (27%) | 143 (52%) | 49 (35%) | 111 (51%) |
| Memory complaints (yes) | 101 (50%) | 123 (45%) | 66 (47%) | 99 (45%) |
| Depression symptoms (0-60) | 19 (12) | 16 (11) | 19 (12) | 16 (10) |
| Visual difficulties (yes) | 96 (52%) | 104 (36%) | 67 (48%) | 81 (37%) |
| Hearing difficulties (yes) | 364 (30%) | 80 (27%) | 38 (27%) | 61 (27%) |
| Chronic diseases (n) |  |  |  |  |
| None | 25 (13%) | 82 (30%) | 20 (15%) | 61 (28%) |
| 1 | 54 (33%) | 77 (28%) | 35 (25%) | 63 (29%) |
| 2 | 56 (24%) | 55 (20%) | 43 (31%) | 45 (21%) |
| 3 or more | 64 (30%) | 59 (22%) | 42 (30%) | 51 (23%) |
| Functional limitations |  |  |  |  |
| None | 35 (18%) | 111 (41%) | 25 (18%) | 87 (40%) |
| Some | 57 (29%) | 80 (29%) | 37 (26%) | 68 (31%) |
| A lot | 107 (54%) | 82 (30%) | 78 (56%) | 65 (30%) |
